# Surgical Resection of Biatrial Myxoma Complicated by Massive Bilateral Pulmonary Tumor Embolism Under Deep Hypothermic Circulatory Arrest: A Case Report

**DOI:** 10.7759/cureus.110901

**Published:** 2026-06-15

**Authors:** Kazuyuki Ishibashi, Mamika Motokawa, Mohammad Moniruzzaman, Aslam Ahmed, Shariful Islam

**Affiliations:** 1 Cardiovascular Surgery, Ship International Hospital, Dhaka, BGD

**Keywords:** benign cardiac tumor, biatrial myxoma, deep hypothermic circulatory arrest, pulmonary artery tumor embolism, right atrial myxoma, tumor morphology, tumor resection

## Abstract

Primary cardiac myxomas are the most common benign cardiac tumors and typically originate in the left atrium. Biatrial myxomas are extremely rare, and their association with massive bilateral pulmonary tumor embolism is even more uncommon. We report the case of a 32-year-old woman who presented with severe dyspnea and hypoxemia. Echocardiography and contrast-enhanced computed tomography revealed a large spherical tumor in the right atrium, a band-like tumor in the left atrium, and extensive tumor emboli obstructing the bilateral main and peripheral pulmonary arteries. The biatrial tumors were resected en bloc with the interatrial septum, and the pulmonary artery tumor emboli were removed under deep hypothermic circulatory arrest. Histopathological examination confirmed that all specimens were myxomas. Postoperatively, the patient’s respiratory symptoms resolved, her estimated pulmonary artery systolic pressure decreased from 90 mmHg to 50 mmHg, and her oxygen saturation improved from 88% to 93% on room air. Although follow-up computed tomography performed three months after surgery demonstrated a residual tumor in the left pulmonary artery, the patient remained asymptomatic under warfarin anticoagulation therapy at the six-month postoperative follow-up. This case highlights a rare presentation of biatrial myxoma with massive bilateral pulmonary tumor embolism, in which intracardiac flow dynamics may have contributed to the distinct tumor morphologies, and demonstrates the utility of deep hypothermic circulatory arrest for complex pulmonary embolectomy.

## Introduction

Cardiac myxomas are the most common primary cardiac tumors, accounting for approximately 50% of all benign cardiac neoplasms. Approximately 75% of these tumors occur in the left atrium (LA), whereas 15%-20% arise in the right atrium (RA); however, myxomas simultaneously involving both atria are extremely rare [1]. Although pulmonary tumor embolism secondary to a right atrial myxoma is uncommon [2], urgent surgical intervention is often required because this condition can lead to life-threatening complications, including severe pulmonary hypertension, profound hypoxemia, and sudden circulatory collapse.

The surgical management of concurrent biatrial myxomas and massive bilateral pulmonary artery tumor embolism associated with severe pulmonary hypertension represents a formidable technical challenge. To achieve complete removal of tumor emboli from the pulmonary arteries, advanced perfusion strategies such as deep hypothermic circulatory arrest (DHCA) may be necessary to provide a bloodless operative field and prevent the distal migration of fragile tumor fragments [3,4].

Herein, we report an unusual case of a 32-year-old woman with biatrial myxomas exhibiting distinct morphologies, with a spherical tumor in the RA and a band-like tumor in the LA, complicated by massive bilateral pulmonary tumor embolism. This case highlights a unique anatomical presentation in which intracardiac flow dynamics may have contributed to the distinct tumor morphologies and demonstrates the safety and utility of single-stage surgical resection under DHCA for complex pulmonary tumor embolectomy.

## Case presentation

A 32-year-old woman presented to our emergency department with progressive dyspnea. Initial evaluation revealed a blood pressure of 110/60 mmHg, a heart rate of 110 beats/min, and an oxygen saturation (SpO₂) of 88% on room air. Physical examination revealed no skin pigmentation, mucocutaneous lesions, or neurological deficits. She had no history of endocrine disorders, and her family history was unremarkable. No deep vein thrombosis (DVT) was detected on the lower extremity venous ultrasound. Laboratory tests revealed a hemoglobin level of 10.5 g/dL (reference range: 13.5 ± 2 g/dL), a white blood cell count of 11.26 × 10³/μL (reference range: 4.0-11.0 × 10³/μL), C-reactive protein of 7.0 mg/L (reference range: <10 mg/L), and an elevated N-terminal pro-brain natriuretic peptide (NT-proBNP) level of 518 pg/mL (reference range: 0-300 pg/mL). The electrocardiogram showed normal sinus rhythm without ischemic changes. Chest X-ray revealed cardiac enlargement with a cardiothoracic ratio of 60% and prominence of the left second cardiac arch; however, the lung fields were clear, with no pleural effusion (Figure 1).　　

**Figure 1:**
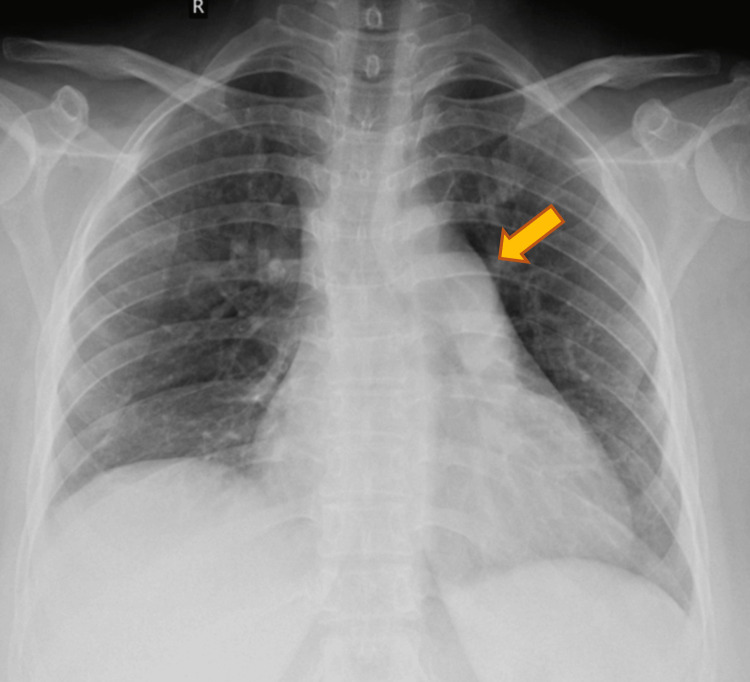
Chest X-ray demonstrating cardiomegaly with a cardiothoracic ratio of 60% and prominence of the second arch on the left cardiac border (arrow), with clear lung fields and no signs of pleural effusion.

Transthoracic echocardiography revealed a large, highly mobile spherical tumor occupying the RA and attached to the interatrial septum (IAS), as well as a mobile band-like tumor in the LA. The estimated pulmonary artery systolic pressure (PASP) was markedly elevated at 90 mmHg. Contrast-enhanced chest computed tomography (CT) confirmed the presence of a large RA tumor, a small LA tumor, several partially obstructing tumor emboli in peripheral pulmonary artery branches, and massive tumor emboli obstructing the bilateral main pulmonary arteries (Figures 2-4).

**Figure 2:**
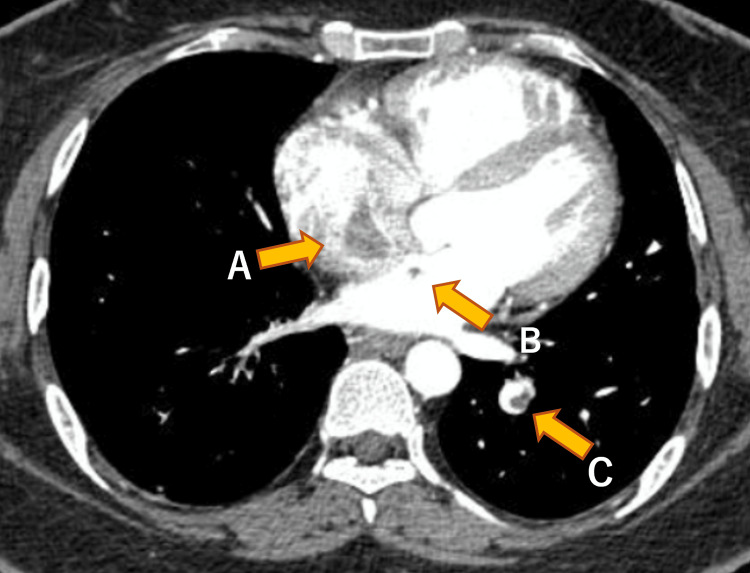
Preoperative contrast-enhanced chest CT (axial view of the atrium). Contrast-enhanced CT reveals a large, spherical tumor shadow within the RA (arrow *A*), smaller tumor components in the LA (arrow *B*), and a filling defect representing a small tumor embolus in the peripheral pulmonary artery (arrow *C*). CT: computed tomography; LA: left atrium; RA: right atrium.

**Figure 3:**
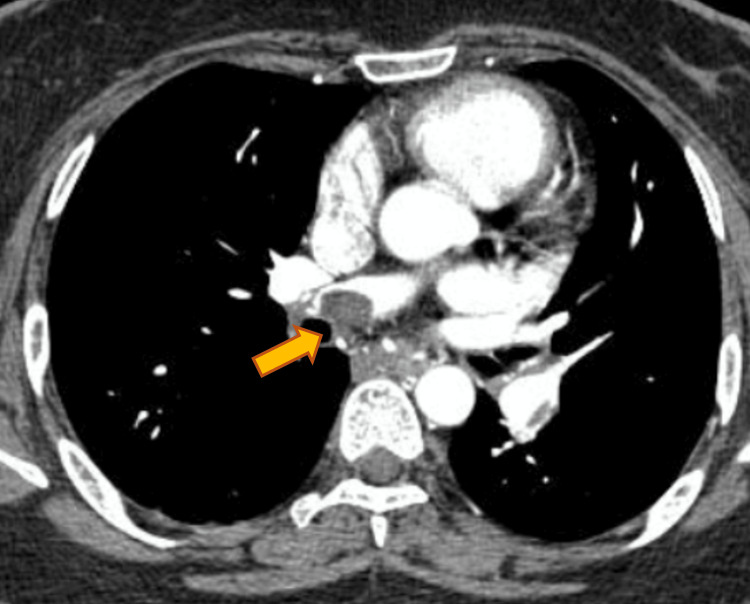
Preoperative contrast-enhanced chest CT (RPA) demonstrating a massive tumor shadow nearly occluding the right main pulmonary artery (arrow). CT: computed tomography; RPA: right pulmonary artery.

**Figure 4:**
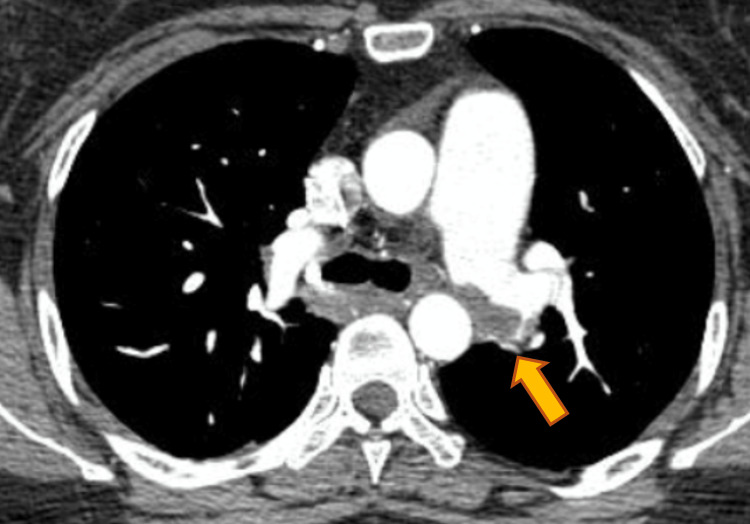
Preoperative contrast-enhanced chest CT (LPA) showing a large tumor shadow partially occluding the left main pulmonary artery (arrow). CT: computed tomography; LPA: left pulmonary artery.

Because of progressive deterioration of respiratory function, a semi-emergent operation was performed through a median sternotomy. Cardiopulmonary bypass (CPB) was established using ascending aortic cannulation and bicaval venous drainage. After aortic cross-clamping and administration of cardioplegia, the RA was opened, revealing an irregular tumor approximately 4 cm in diameter attached to the IAS. First, the right atrial tumor was resected, followed by en bloc resection of the IAS and a band-like left atrial tumor. The patient had neither a patent foramen ovale nor an atrial septal defect. Intraoperative macroscopic examination confirmed that the stalks of both tumors were located superior to the fossa ovalis, fully penetrating the portion of the IAS to demonstrate mutual continuity. The atrial septal defect was reconstructed using an autologous pericardial patch (Figure 5).

**Figure 5:**
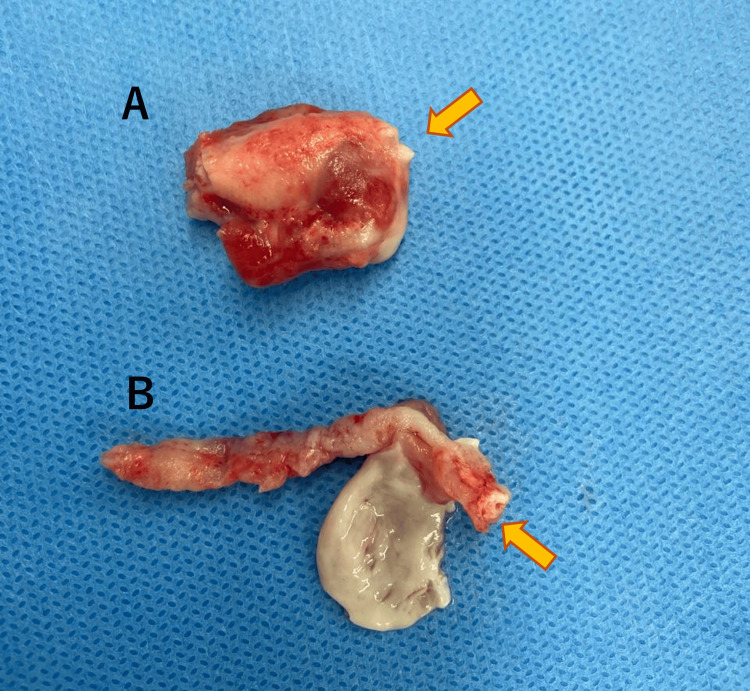
Macroscopic findings of the resected biatrial tumors following surgical manipulation. The right atrial component (A) is spherical with an irregular and fragile surface. The left atrial component (B) is elongated and band-like. The stalks of both specimens were macroscopically continuous through the interatrial septum. The areas indicated by arrows (in A and B) were originally connected but became separated during surgical manipulation.

To safely clear the extensive pulmonary emboli, the patient was cooled slowly to a rectal temperature of 25°C to DHCA. During the circulatory arrest periods, topical head cooling with ice was maintained for cerebral protection. An incision was extended from the main pulmonary artery into the left pulmonary artery (LPA). To extract the tumor fragments without leaving residual tissue or damaging the arterial wall, the tumor was carefully dissected and peeled away from the vascular intima using a slender, blunt-tipped suction cannula along with forceps. Because the LPA tumor was extremely fragile and difficult to grasp, it was carefully extracted using suction and forceps. After 12 minutes of circulatory arrest, reperfusion was performed for 10 minutes while the LPA was closed. A second period of circulatory arrest was then initiated, and the right pulmonary artery (RPA) was incised between the ascending aorta and superior vena cava. The RPA tumor was successfully removed en bloc. The second circulatory arrest time was 8 minutes (Figure 6). After closure of the RPA and RA, the patient was successfully weaned from CPB without difficulty.

**Figure 6:**
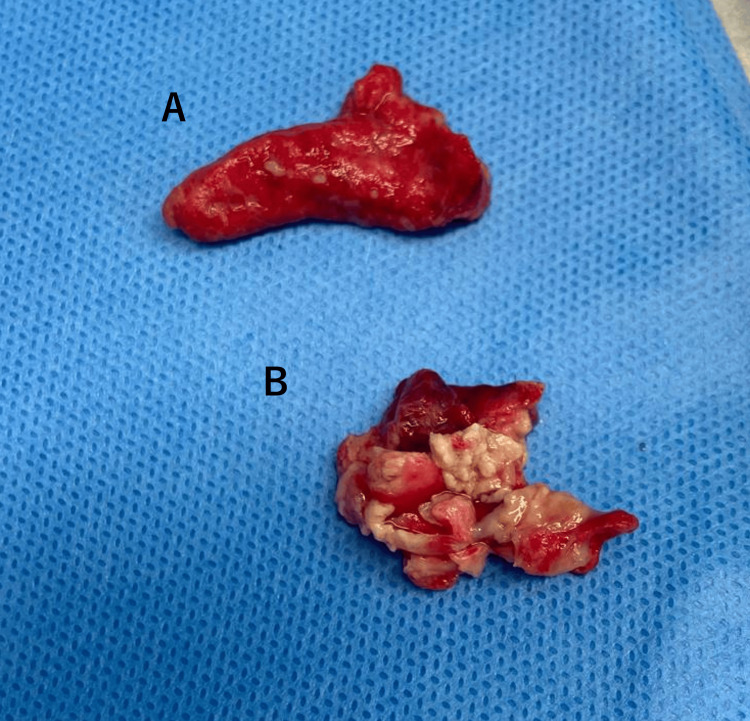
Macroscopic findings of the resected pulmonary tumor emboli. The specimen from the RPA (A) exhibits a relatively smooth surface, whereas the tissue from the LPA (B) is gelatinous and extremely friable. RPA: right pulmonary artery; LPA: left pulmonary artery.

Histopathological examinations in all four specimens, spindle-shaped and polygonal cells were observed scattered within a myxedema-like matrix. The cells were surrounded by abundant eosinophilic cytoplasm and had small nuclei; no nuclear atypia was observed, leading to a diagnosis of atrial myxoma. Additionally, thrombi were observed in a portion of the excised tissue. No malignant features, such as mitotic figures or necrosis, were observed in the specimens. The patient was extubated on postoperative day 1 and discharged on postoperative day 7 without respiratory symptoms. At discharge, her room-air SpO₂ had improved to 93%, and the estimated PASP had decreased to 50 mmHg. Although follow-up CT performed three months postoperatively demonstrated residual or recurrent tumor within the LPA (Figure 7), the patient remained asymptomatic under warfarin anticoagulation therapy at the six-month follow-up, with an SpO₂ of 94% on room air.

**Figure 7:**
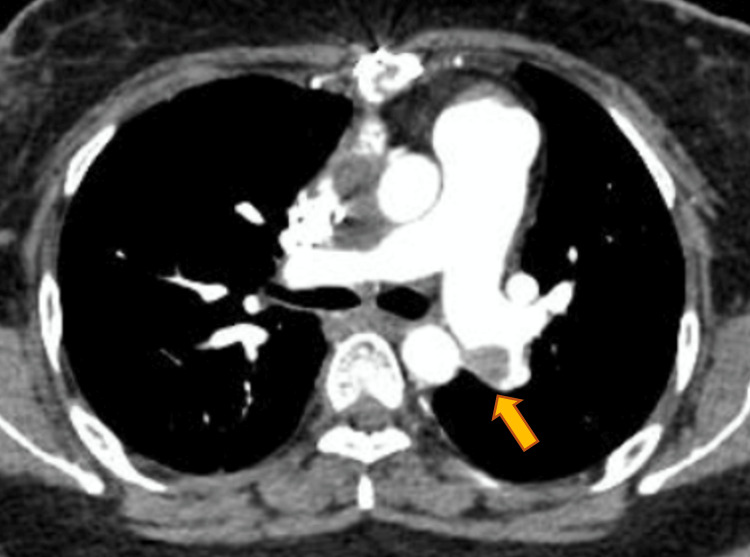
Follow-up contrast-enhanced chest CT at three months postoperatively. No residual tumor shadow is observed in the RPA, whereas a persistent tumor shadow (residual or recurrent fragment) is detected in the LPA (arrow). CT: computed tomography; RPA: right pulmonary artery; LPA: left pulmonary artery.

## Discussion

Biatrial cardiac myxoma is a rare clinical entity. In most reported cases, the tumor demonstrates a “butterfly” or “dumbbell-shaped” configuration traversing the fossa ovalis, with similar spherical or oval masses on both sides [5,6]. In contrast, this case demonstrated a striking morphological disparity, consisting of a large spherical tumor in the RA and an elongated band-like tumor in the LA.

From a hemodynamic perspective, this morphological difference may be attributable to the distinct intracardiac pressure and flow characteristics of each chamber. The RA is a low-pressure, low-velocity chamber that provides a relatively stagnant environment, potentially allowing the tumor to expand uniformly into a spherical configuration. In contrast, the LA is exposed to higher pressure and more rapid, directional blood flow, which may mechanically constrain tumor growth and contribute to the formation of an elongated or rod-like structure. Although cardiac myxomas are macroscopically classified into “round” (solid) and “gelatinous” (papillary) types [5], few reports have discussed the potential influence of intracardiac hemodynamic forces on tumor morphology during growth.

The most life-threatening factor in this case was bilateral pulmonary tumor embolism. Left-sided myxoma often causes systemic embolism [7], but pulmonary tumor embolism secondary to right-sided myxoma is uncommon [8], and bilateral main pulmonary artery occlusion is extremely rare [4]. Ran et al. described a remarkable case of a biatrial myxoma that simultaneously triggered both pulmonary and cerebral embolism, demonstrating the unpredictable and multi-directional nature of myxomatous embolization [9]. In this patient, the RA tumor exhibited a fragile and irregular surface morphology. We hypothesize that mechanical shear stress fragmented the tumor surface, leading to embolism in both pulmonary arteries. These embolic fragments then likely promoted local thrombus formation and tumor growth within the pulmonary arteries, ultimately resulting in partial vascular occlusion. Furthermore, multiple peripheral pulmonary artery embolism are thought to have contributed to widespread ventilation-perfusion mismatch, resulting in severe pulmonary hypertension (estimated PASP 90 mmHg) and severe hypoxemia. When a patient presents with large filling defects in both main pulmonary arteries accompanied by severe pulmonary hypertension, the differential diagnosis should primarily include acute thromboembolism and pulmonary artery sarcoma (PAS).

Acute pulmonary thromboembolism is the most common clinical entity and presents with identical initial symptoms, such as progressive dyspnea and severe hypoxemia. However, given the absence of DVT and the concurrent presence of highly mobile masses in both atria, a tumor-induced embolic event is strongly suspected rather than typical thromboembolism secondary to DVT. On CT, distinguishing between a benign myxoma embolism and a highly malignant PAS is extremely difficult unless extravascular invasion is present. In recent years, magnetic resonance imaging (MRI) has emerged as a preferred modality for differentiating PAS from acute or chronic thromboembolic disease; pulmonary sarcomas typically exhibit enhancement with gadolinium contrast agents, whereas thrombi do not. Furthermore, fluorodeoxyglucose positron emission tomography (FDG-PET) has become a valuable technique for diagnosing and staging, as increased FDG uptake can confirm tumor malignancy and identify distant metastases [10]. In this case, a definitive diagnosis was ultimately established through a thorough histopathological evaluation of the specimens resected from both the heart and the pulmonary artery, which revealed classic myxoma tissue with adjacent appositional thrombus and no signs of cellular malignancy.

Furthermore, distinguishing between residual/recurrent tumor fragments and normal blood thrombi is clinically crucial, particularly regarding the lesion detected in the left pulmonary artery on the follow-up CT scan three months postoperatively. Considering that the left pulmonary artery tumor was extremely friable and was removed fragmentarily during surgery, this postoperative shadow could represent either a true residual myxoma fragment or an adherent thrombus formed at the surgical site. While typical acute thrombi often resolve completely with standard anticoagulation, myxomatous emboli carry a persistent risk of local progression and distant metastasis. Therefore, anticoagulation therapy with warfarin is being continued alongside ongoing radiographic surveillance to monitor for any progressive luminal changes.

Complete surgical extraction of fragile tumor emboli from the distal pulmonary arterial tree requires an optimal operative field. We selected DHCA to achieve a completely bloodless field, enabling safe visualization and removal of the fragile myxomatous tissue from the bilateral pulmonary arteries while minimizing the risk of distal embolic migration [7]. Although the right pulmonary artery (RPA) tumor was successfully removed en bloc, the left pulmonary artery (LPA) lesion was extremely friable, making complete extraction technically difficult. The residual or recurrent lesion identified on follow-up CT may therefore reflect incomplete removal of the fragile embolic material. This finding underscores the technical challenges associated with pulmonary tumor embolectomy for myxomatous emboli and highlights the importance of strict long-term radiographic surveillance.

Finally, the presence of multiple or biatrial myxomas raises suspicion for Carney complex, an autosomal dominant syndrome characterized by spotty skin pigmentation, cardiac and extracardiac myxomas, and endocrine overactivity [11]. However, our patient exhibited no mucocutaneous abnormalities, endocrine disorders, or relevant family history suggestive of this syndrome. Therefore, this case was considered a sporadic form of biatrial myxoma, making its unusual anatomical morphology and embolic presentation even more noteworthy.

## Conclusions

We successfully treated a rare case of biatrial myxoma presenting with different morphologies as a spherical tumor in the RA and a band-like tumor in the LA. This patient was complicated by severe pulmonary hypertension and hypoxemia due to massive bilateral pulmonary tumor embolism. Single-stage surgical resection using DHCA proved to be a safe and effective strategy for pulmonary tumor embolectomy and management of the biatrial lesions. The marked morphological differences between the right and left atrial tumors may reflect the different intracardiac hemodynamic environments of each chamber. Because pulmonary tumor embolisms are extremely fragile and often difficult to completely remove, careful long-term postoperative follow-up with imaging studies is essential to detect residual lesions and recurrence.
